# Microwave-assisted hydrothermal synthesis of amino acid-loaded Cu_2_O hybrid particles for CO_2_ reduction electrocatalysis†

**DOI:** 10.1039/d5ra02252e

**Published:** 2025-05-15

**Authors:** Yuki Tsuda, Mizuki Irizawa, Saki Fukuma, Minami Kato, Takao Gunji, Kazuki Yoshii, Nobuhiko Takeichi

**Affiliations:** a Research Institute of Electrochemical Energy (RIECEN), Department of Energy and Environment, National Institute of Advanced Industrial Science and Technology (AIST) Ikeda Osaka 563-8577 Japan y-tsuda@aist.go.jp; b Renewable Energy Advanced Research Center (READ), National Institute of Advanced Industrial Science and Technology (AIST) 2-2-9 Machiikedai, Koriyama Fukushima 963-0298 Japan; c Department of Chemical and Environmental Engineering, The University of Kitakyushu Kitakyushu Fukuoka 808-0135 Japan

## Abstract

Amino acid-loaded Cu_2_O hybrid particles were synthesized *via* microwave-assisted hydrothermal reaction for efficient CO_2_ reduction. The amino acid-loaded Cu_2_O particles exhibited different selectivities in the CO_2_ electrolysis products depending on type of loaded amino acid. Notably, as compared to amino acid-unloaded Cu_2_O particles, the l-histidine-loaded Cu_2_O hybrid particles exhibited an improvement of faradaic efficiency to 18.5% toward ethylene production.

## Abstract 2

Amino acid-loaded Cu_2_O hybrid particles were synthesized *via* microwave-assisted hydrothermal reaction for efficient CO_2_ reduction.

## Graphical abstract

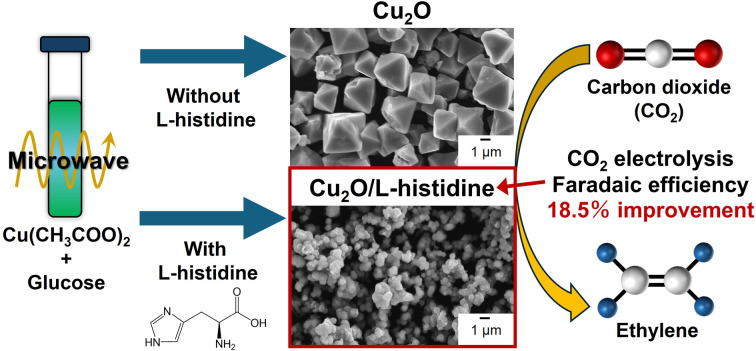

## 1 Introduction

The increasing concentration of carbon dioxide (CO_2_) in the atmosphere is a critical environmental issue, because of its significant contribution to global warming and climate change.^1,2^ Efforts to mitigate CO_2_ emissions have led to research on various strategies, including carbon capture and storage, utilization of renewable energy sources, and development of technologies for CO_2_ conversion and utilization.^3^ Among the researched CO_2_ mitigation strategies, electrochemical CO_2_ reduction has garnered substantial attention as a promising approach to reducing atmospheric CO_2_ levels.^4^ Electrochemical CO_2_ reduction offers several advantages, including the ability to operate under mild conditions, the potential to integrate with renewable energy sources, and the possibility to produce a wide range of value-added products. However, the efficiency and selectivity of the CO_2_ reduction reaction (CO_2_RR) process are highly dependent on the type of the used catalyst. To improve the product selectivity of CO_2_ electrolysis, the competing hydrogen evolution reaction (HER) must be minimized or suppressed. Therefore, developing effective electrocatalysts that can efficiently convert CO_2_ into desired products *via* electrolysis while minimizing the unwanted HER is crucial for advancing the CO_2_ electrolysis technology.

Recent advances in the design of catalysts for the electrochemical CO_2_ reduction have focused on improving their activity, selectivity, and stability. Various materials, including metals,^5^ metal oxides,^6^ metal–organic frameworks,^7^ and molecular composites,^8^ have been investigated for their potential to facilitate CO_2_ reduction. Among the catalysts studied for CO_2_ reduction, copper-based catalysts have been reported as the catalysts that facilitate CO_2_ reduction to produce hydrocarbons and alcohols; however, high overpotentials and low product selectivity are the challenges associated with the use of copper-based catalysts.^9^ Cuprous oxide (Cu_2_O) is a particularly promising catalyst for CO_2_ electrolysis, as it facilitates the production of ethylene (C_2_H_4_), an important industrial chemical.^10^ The selectivity of CO_2_ electrolysis for producing the desired products depends on the adsorptive power of reaction intermediates, such as carbon monoxide (CO), on the catalyst.^11^ To control the adsorptive power of reaction intermediates, controlled physicochemical modifications, such as alloying^12^ and decorating inorganic catalysts using organic materials,^13^ have been explored. Previously, we explored the performance of electrodeposited Cu loaded with different types of amino acids toward electrocatalytic CO_2_RR.^14^ We tested the product selectivities of five different electrodeposited Cu-based catalysts during CO_2_ electrolysis. The faradaic efficiency (FE) of methane (CH_4_) production *via* CO_2_ electrolysis was found to depend on the type of amino acid loading in the electrodeposited Cu. The electrodeposited Cu without any amino acid produced CH_4_ with an FE of 55.0%, and the electrodeposited Cu loading l-histidine, a type of imidazole-containing amino acid, produced CH_4_ with an FE of 67.6%.^14^ In conclusion, introducing organic components into inorganic catalysts is an effective strategy for improving their product selectivities during CO_2_ electrolysis.

**Fig. 1:**
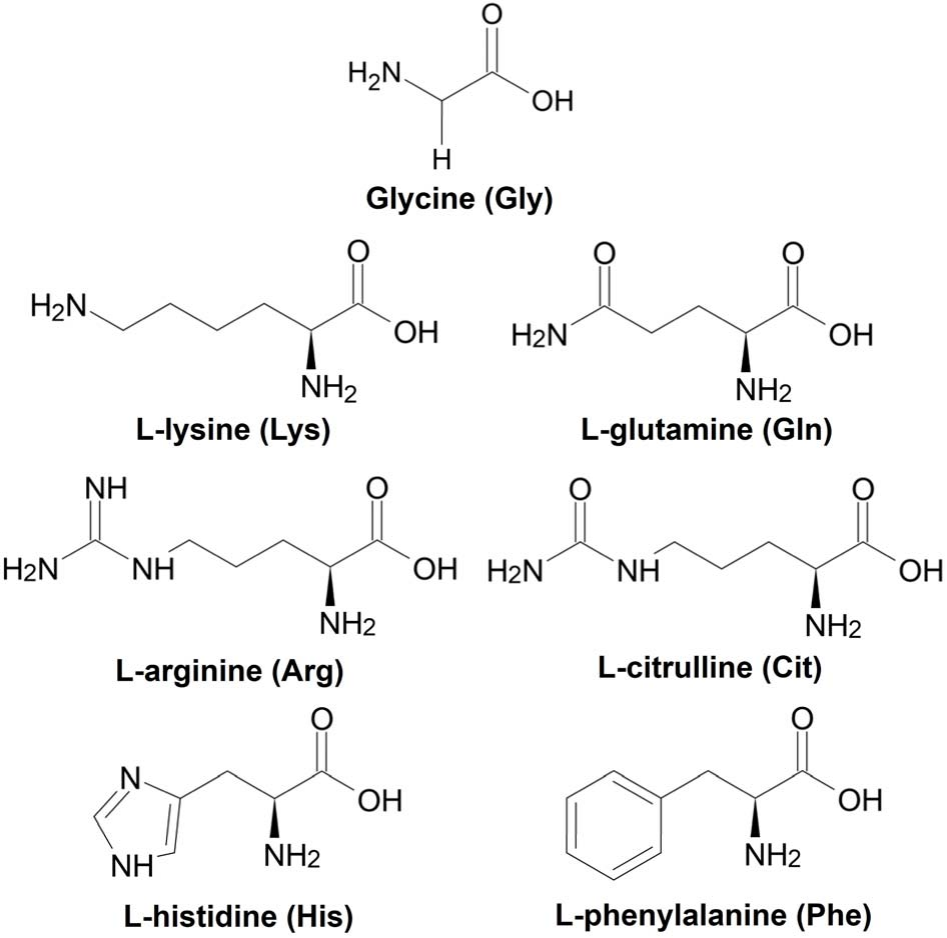
Structures of the amino acids used is this study.

Hydrothermal synthesis is one of the promising methods for synthesizing functional materials because it can provide control over the crystal orientation, particle size, and particle shape by adjusting the synthesis temperature and reaction time, by changing material source and precursor concentrations, and by adding structure-directing agents (SDAs).^15^ Furthermore, the reactions involved in hydrothermal synthesis can be accelerated by microwave radiation and homogeneous heating, resulting in synthesizing high-purity crystals.^16^ In this study, we synthesized hybrid particles of Cu_2_O loading seven different amino acids (Fig. 1)—Glycine (Gly), l-lysine (Lys), l-glutamine (Gln), l-arginine (Arg), l-citrulline (Cit), l-histidine (His), or l-phenylalanine (Phe)— using a microwave-assisted hydrothermal method and evaluated their product selectivities during CO_2_ electrolysis. Loading amino acids into Cu_2_O particles can be anticipated to alter the adsorption of reaction intermediates on their surfaces, affecting their product selectivities during CO_2_ electrolysis.

## 2 Experimental

### 2.1 Materials

Copper (II) acetate (Cu(CH_3_COO)_2_, 97.0%, Wako), d(+)-glucose (C_6_H_12_O_6_, 98.0%, Wako), glycine (Gly, C_2_H_5_NO_2_, 99.0%, Wako), l-lysine (Lys, C_6_H_14_N_2_O_2_, 95.0%, Wako), l-glutamine (Gln, C_5_H_10_N_2_O_3_, 99.0%, Wako), l-arginine (Arg, C_6_H_14_N_4_O_2_, 98.0%, Wako), l-citrulline (Cit, C_6_H_13_N_3_O_3_, 97.0%) l-histidine (His, C_6_H_9_N_3_O_2_, 98.0%, Wako), l-phenylalanine (Phe, C_9_H_11_NO_2_, 99.0%, Wako) and potassium hydrogen carbonate (KHCO_3_, 99.5%, Wako) were purchased and used as received without further purification. Structures of amino acids used in this research showed in Fig. 1.

### 2.2 Synthesis of amino acids-loaded Cu_2_O hybrid particles

Aqueous precursor solutions containing 0.2 mol dm^−3^ Cu(CH_3_COO)_2_, 0.1 mmol dm^−3^d(+)-Glucose, and 5.0 mmol dm^−3^ of amino acids was put in glass vial, set to a Biotage® Initiator + Robot Eight for a 2.45 GHz microwave radiation to promote reaction at 130 °C for 10 min. The resulting samples were centrifugally separated at 4500 rpm for 10 min, washed with ultrapure water and ethanol each for 3 times, and dried in an oven at 60 °C for 12 h under vacuum condition.

### 2.3 Characterizations

The crystal structures were determined by X-ray diffraction (XRD) spectroscopy with a scan range of *θ*–2*θ* scans using a RIGAKU Ultima IV X-ray diffractometer. The average crystallite size of the synthesized samples was calculated from the Cu_2_O (111) diffraction peak, the most intense of the four peaks, using the Scherrer equation eqn (1):1
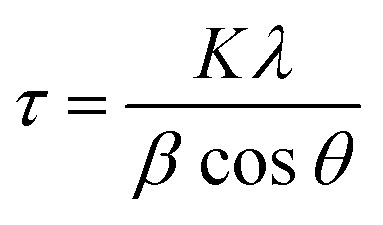
where *τ*, *K*, *λ*, *β*, and *θ* represent the average crystallite size, shape factor (≈0.9), X-ray wavelength (CuKα ≈ 1.54 Å), full width at half maximum, and Bragg angle, respectively. Scanning electron microscopy (SEM) measurements were conducted using a JEOL JSM-IT100. The X-ray photoelectron spectroscopy (XPS) measurements were conducted using a PHI 5000 VersaProbe (ULVAC-PHI, Japan) with monochromatic Al Kα X-rays (1486.6 eV). The X-ray irradiation output was set to 25 W at 15 kV. In this process, the peak calibration was performed using the binding energy of C1s, set at 284.6 eV. High-angle annular dark-field scanning transmission electron microscopy (HAADF-STEM) and elemental mapping images were acquired by energy dispersive X-ray spectroscopy (EDS) using a Talos F200X G2 (Thermo Fisher Scientific) equipped with ADF-STEM systems and operated at 200 kV. Thermogravimetric (TG) analysis was performed by DTG-60 SHIMADZU. BET specific surface area was measured on a BELSORP MAX analyzer (MicrotracBEL, Japan) under liquid N_2_ temperature conditions (77 K). Prior to BET measurements, deaeration was conducted at 80 °C for 10 h. The loading density of amino acid (*L*, mol g m^−2^) was calculated by weight reduction (Δ*m*, g) from TG analysis, molecular weight (*M*, mol L^−1^), and specific surface area (*S*, m^2^ g^−1^) from BET measurements following eqn (2).2
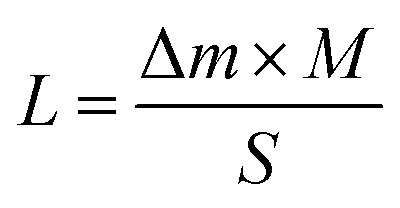


Fourier transform infrared (FT-IR) spectra were measured by SHIMADZU IRTracer-100 with attenuated total reflection (ATR) unit.

### 2.4 Preparation of electrodes

For electrochemical measurements, synthesized particles were processed into electrodes. The particles (10 mg) were sufficiently dispersed in methanol (500 mg, Wako 99.5%) with Nafion ionomer (80 mg, 5 wt%, Sigma-Aldrich) by using ultrasonic equipment. The resulting slurry was drop-casted onto carbon paper (CP, SGL carbon, SIGRACET®), then dried at 70 °C for 12 h under vacuum condition. Prior to drop-casted, the CP was cut into pieces measuring 10 × 20 mm. The projected area of the working electrode was confined to 1.0 cm^2^. This confinement was achieved using polyimide tape.

### 2.5 Electrochemical CO_2_ reduction

The evaluation of the catalytic activities of electrochemical CO_2_ reduction was conducted in a simple three electrodes H-type cells containing CO_2_ saturated 13 mL aqueous KHCO_3_ solution (pH ≈ 8.75) using an HZ-Pro S12 electrochemical measurement system (HOKUTO DENKO). For CO_2_ saturating in electrolytic bath, the solution was continuously bubbled with highly pure CO_2_ (99.995%) at a flow rate of 20 mL min^−1^ for 20 min before conducting electrolysis. In the electrolytic cell, prepared each electrode, a silver–silver chloride (Ag/AgCl) electrode (3 mol dm^−3^ KCl), and Pt wire were used as the working, reference, and counter electrodes, respectively. The cathode and anode were separated by anion exchanging membrane (ASTOM Corporation, NEOSEPTA AHA). The CO_2_ electrolysis was conducted by constant potential electrolysis at −1.27 V *vs.* reversible hydrogen electrode (RHE) to ensure a charge of 3.0 C. The potential of *vs.* Ag/AgCl was converted to the RHE scale using eqn (3):

3 *E*_RHE_ = *E*_Ag/AgCl_ + 0.0591 × pH + 0.208

The gaseous products of CO_2_ electrolysis, including CO_2_ and H_2_, were analyzed using a gas chromatograph (GC) equipped with both a mass spectrometer (GCMS-QP2010 Ultra, SHIMADZU) and a thermal conductivity detector (GC-8AIT, SHIMADZU). 0.1 mL sample of the produced gas from the 12 mL headspace of the cell was extracted using a gas-tight syringe and then injected into the GC system for detailed compositional analysis. The CO_2_ electrolysis products analysis was conducted at least three times in each condition.

The faradaic efficiency (FE) of gaseous products were calculated by following eqn (4),4
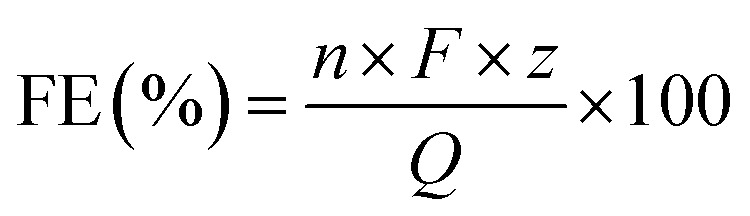
in which *n*, *F*, *z*, and *Q* represents the amount of moles of the product (mol), the Faraday's constant (96 485 C mol^−1^), the number of electrons transferred per molecule of the product, and the total charge passed during the electrolysis (C), respectively.

## 3 Results and discussion

**Fig. 2:**
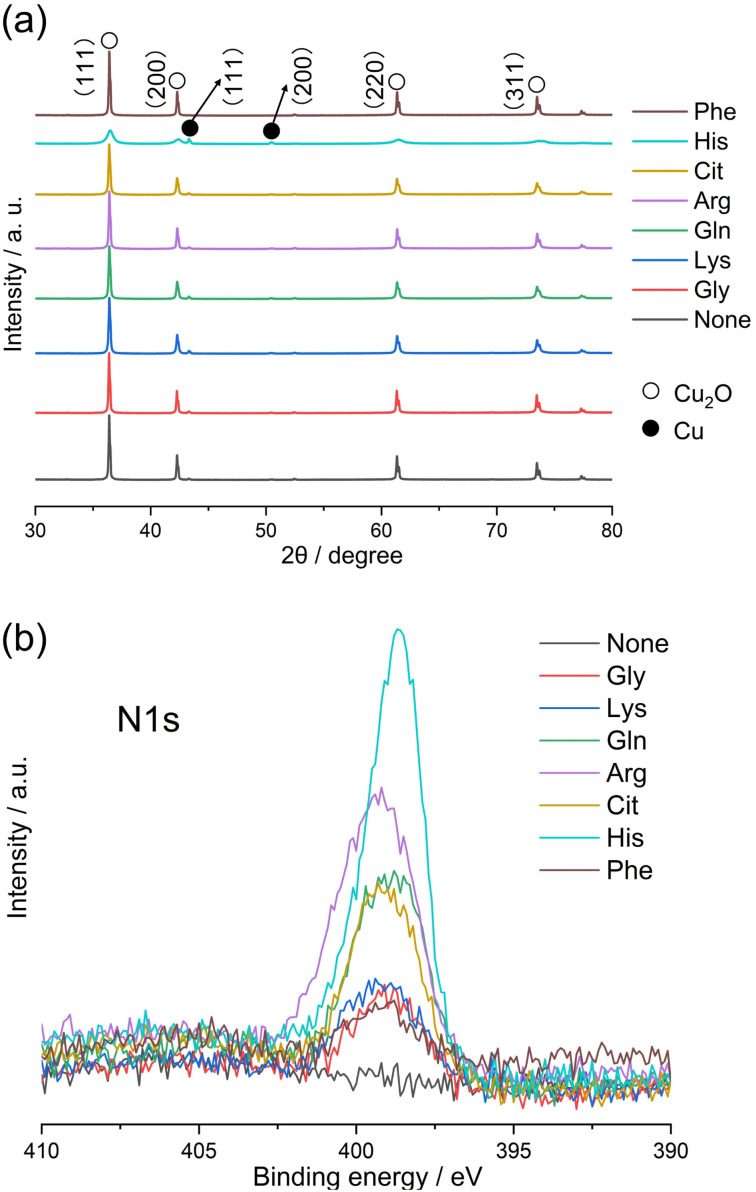
(a) XRD patterns and (b) N1s core-level XPS spectra of the Cu_2_O particles synthesized with 5.0 mmol dm^−3^ amino acids.

**Fig. 3:**
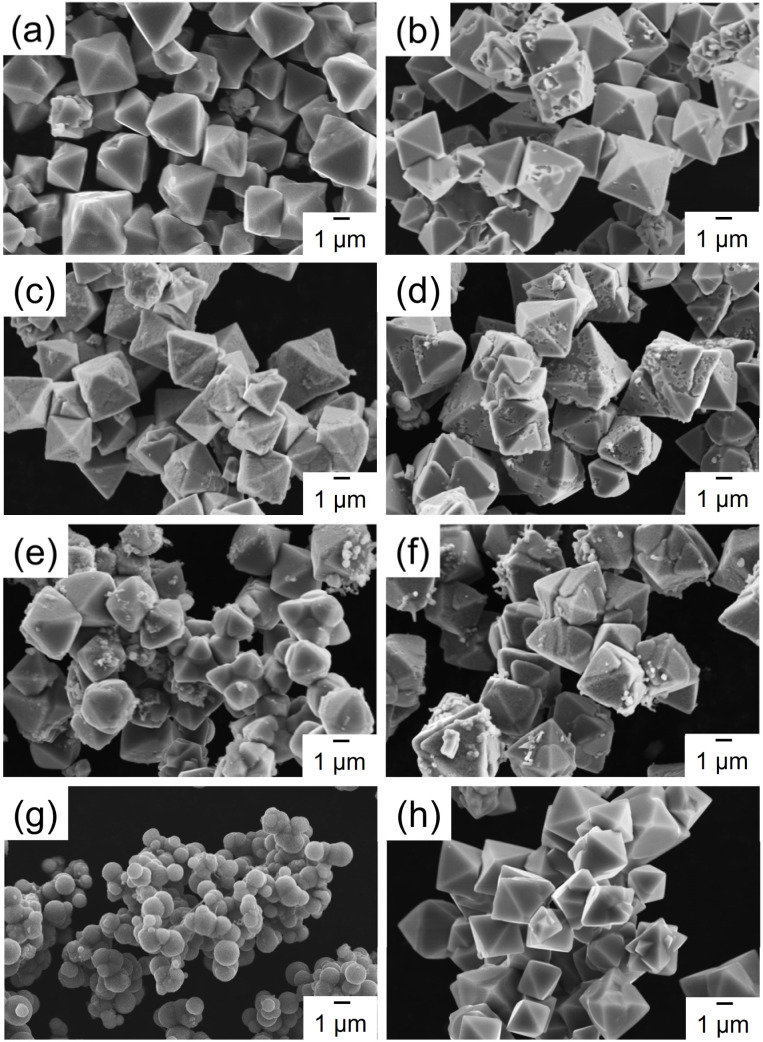
SEM images of the Cu_2_O particles synthesized with 5.0 mmol dm^−3^ amino acids: (a) None, (b) Gly, (c) Lys, (d) Gln, (e) Arg, (f) Cit, (g) His, and (h) Phe.

Gly has a standard amino acid structure, containing both carboxyl and amino groups. Lys, Arg, and His are basic amino acids, whereas Gln, Cit, and Phe are neutral amino acids with similar structures. The synthesis of powdered samples, irrespective of the presence or absence of amino acids, was achieved *via* a hydrothermal process using microwave radiation. Indian red-colored powder samples were obtained; however, an orange-colored powder was obtained when l-His was used. The difference in the observed colors of the particles can be attributed to their different of the particle sizes, as discussed later. XRD patterns of the Cu_2_O powders synthesized with different amino acids or without amino acids (hereafter referred to as None) are shown in Fig. 2a. The XRD patterns in Fig. 2a show peaks at approximately 36.4°, 42.3°, 61.4°, and 73.5°, corresponding to the (111), (200), (220), and (311) planes in Cu_2_O, respectively. XRD analysis also reveals the presence of low intensity peaks at 43.4° and 50.5°, attributed to the (111) and (200) planes in Cu, respectively (Fig. S1†). Note that His possesses an imidazole group and exhibits high electron-donating ability, functioning as a reducing agent.^17^ In this experiment, the relatively stronger diffraction peak intensity exhibited by the His-loaded Cu_2_O particles can be attributed to the reducing property of His. The Cu_2_O particles synthesized with different amino acids, excluding those loaded with His, exhibit XRD patterns similar to that of None, with no observable changes attributable to the loaded amino acids. The presence of His in the Cu_2_O particles decreases the intensities and increases the widths of the Cu_2_O-related peaks. The average crystallite sizes in the synthesized Cu_2_O particles were calculated by applying the Scherrer equation on the (111) diffraction peak of Cu_2_O, and the results are shown in Fig. S2.† The crystallite sizes in None, Gly, and Phe are 916, 912, and 926 Å, respectively, which are almost identical. The crystallite size in Arg is 846 Å, which is slightly smaller than that in None. The crystallite sizes in Lys, Gln, and Cit are 730, 713, and 734 Å, respectively, which are smaller than those in Arg. The crystallite size in His is 147 Å, which is the lowest value among all studied samples. To validate the loading of amino acids into Cu_2_O particles, N1s XPS spectra were measured for the Cu_2_O powders synthesized with different amino acids. Amino acid-loaded Cu_2_O hybrid particles should exhibit a peak in the N1s core-level XPS spectrum, indicating the loading of organic components. In Fig. 2b, no peaks can be observed in the N1s XPS spectrum corresponding to None, whereas a peak can be observed in the XPS spectra of the Cu_2_O particles synthesized with amino acids. The observed differences in the XPS peak shapes originate from the different functional groups present in the amino acids. Note that the functional groups (or side chain) of amino acids determine their chemical properties and how they interact with each other and their environment. Notably, the amino acids used in this study would not undergo decomposition while synthesizing Cu_2_O particles at a temperature of 130 °C because thermal decomposition of the utilized amino acids occurs in the temperature range of 185–280 °C.^18^ The surface morphologies of the synthesized Cu_2_O particles were examined using SEM, and the results are shown in Fig. 3. SEM images show that None contains octahedron-shaped particles with sizes ranging from 3 to 5 μm (Fig. 3a), whereas Gly (Fig. 1b) and Phe (Fig. 3h) loaded Cu_2_O particles having morphologies similar to that of None. Lys (Fig. 3c), Gln (Fig. 3d), Arg (Fig. 3e), and Cit (Fig. 3f) loaded Cu_2_O particles exhibit slightly deformed shapes with rounded appearances, which are more pronounced for Cit and Arg loaded Cu_2_O particles. Notably, the shape of Cu_2_O particles changes by a relatively greater extent upon His loading. The His-loaded Cu_2_O hybrid particles exhibit a spherical shape, with a radius of approximately 1 μm (Fig. 3g).

**Fig. 4:**
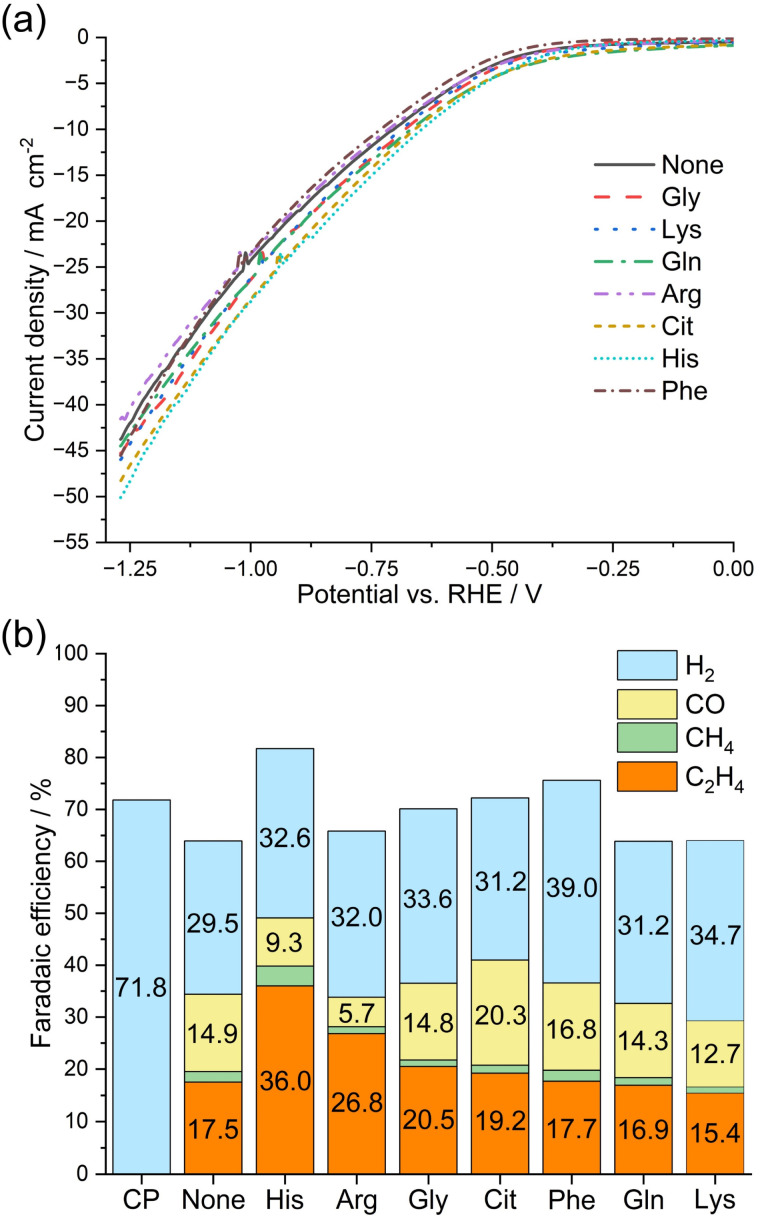
(a) LSV curves of the Cu_2_O-based electrodes synthesized with 5.0 mmol dm^−3^ amino acids at 10 mV s^−1^ in a CO_2_-purged 0.5 mol dm^−3^ aqueous KHCO_3_ solution (pH ≈ 8.75). (b) FE values exhibited by the amino acid-loaded Cu_2_O-based electrodes toward gaseous products during CO_2_ electrolysis under an applied potential of −1.27 V *vs.* RHE at 3.0 C.

The catalytic activity and product selectivity of the synthesized amino acid-loaded Cu_2_O hybrid particles during electrochemical CO_2_RR were evaluated. Initially, the linear sweep voltammogram (LSV) curves were obtained for the synthesized amino acid-loaded Cu_2_O electrocatalyst in a CO_2_-purged 0.5 mol dm^−3^ aqueous KHCO_3_ solution (pH ≈ 8.75), and the results are shown in Fig. 4a. During LSV measurements, no notable differences occur between the electrodes despite the different morphologies and particle sizes of the different amino acid-loaded Cu_2_O catalysts. In the LSV curves illustrated in Fig. 4a, the current can be observed to rise at approximately −0.4 V *vs.* RHE, and as the potential sweeps to negative, the current can be observed to decrease with almost identical slopes for all the investigated catalytic systems. CO_2_ electrolysis was conducted under a constant potential of −1.27 V *vs.* RHE at 3.0 C in a CO_2_-purged 0.5 mol dm^−3^ aqueous KHCO_3_ solution. The chronoamperograms that were measured during CO_2_ electrolysis and the FE values corresponding to the gaseous products obtained from CO_2_ electrolysis are presented in Figs. S3† and 4b, respectively. CP, which was prepared using a Nafion ionomer electrode and served as a reference, showed an electrolytic current of approximately −23 mA cm^−2^. The synthesized Cu_2_O based electrodes shows higher current density than CP. Fig. 4b shows that all the synthesized Cu_2_O electrocatalysis produced H_2_, CO, CH_4_, and C_2_H_4_ after CO_2_ electrolysis. Furthermore, Fig. 4b shows that all the synthesized amino acid-loaded Cu_2_O electrocatalyst facilitate the electrochemical production of H_2_ with an FE of approximately 30% and exhibit an extremely low FE toward the electrochemical production of CH_4_. Notably, the FE values exhibited by the synthesized amino acid-loaded Cu_2_O electrocatalyst toward the production of C_2_H_4_ are comparable to or higher than that exhibited by the None-based electrode (17.5%). The Gly, Cit, Phe, Gln, and Lys-based electrodes produced C_2_H_4_ with FE values of 20.5, 19.2, 17.7, 16.9, and 15.4%, respectively, which are comparable to that obtained using the None-based electrode. Notably, the His and Arg-based electrodes facilitate the production of C_2_H_4_ with high FE values of 36.0 and 26.8%, respectively. Especially, the His-based electrode facilitates the production of C_2_H_4_ with an FE value, which is higher by 18.5% than that exhibited by the None-based electrode. The improvement observed in the production of C_2_H_4_ demonstrated no correlation with the presence of Cu (Fig. S1†) or the average crystallite size (Fig. S2†) within the catalyst. The FE values exhibited by the electrodes toward C_2_H_4_ production are not related to whether the loaded amino acids are basic or neutral, as the Lys-based electrode showed no improvement over the None-based electrode. The FE values toward CO production exhibited by the None, His, Arg, Gly, Cit, Phen, Gln, and Lys-based electrodes are 14.9, 9.3, 5.7, 14.8, 20.3, 16.8, 14.3, and 12.7%, respectively. The surface morphologies and XRD patterns of the electrodes fabricated using the Cu_2_O particles synthesized with 0 and 5.0 mmol dm^−3^ His were examined before and after CO_2_ electrolysis (Fig. S4†). The XRD peak intensities can be observed to remain unchanged even after electrolysis (Fig. S4a†). The surface morphologies of the electrode can be also observed to remain unchanged even after electrolysis (Fig. S4b–e†). The results in Fig. S4,† confirm that the catalysts —Cu_2_O particles containing 0 and 5.0 mmol dm^−3^ His— on the fabricated electrodes remain unchanged during CO_2_ electrolysis. When using the His-loaded Cu_2_O electrocatalysts, the enhanced selectivity of CO_2_ electrolysis toward C_2_H_4_ formation can be associated with two plausible factors: (i) morphology and size control of Cu_2_O particles; and (ii) the effect of loading His into Cu_2_O. First, the morphology of Cu_2_O particles changes from octahedral to spherical upon His loading, forming spherical-shaped His-loaded Cu_2_O hybrid particles. In general, octahedral-shaped Cu_2_O predominantly exposes the (111) facet, which is known to facilitate proton adsorption and reduction, thereby resulting in the formation of formic acid and CO.^19^ Notably, spherical-shaped His-loaded Cu_2_O particles randomly expose various crystal facets across the surface. As a result, the reactivity tends to become uniform while, the selectivity for specific products decreases.^19^ In addition, as the particle size decreases, the specific surface area increases, resulting in a larger surface area involved in the reaction. Therefore, the observed increase in the reaction rate can be anticipated to result from the high catalytic activity of His-loaded Cu_2_O electrocatalysts, making it particularly useful for promoting CO production.^20^ However, due to the presence of any defect sites, the reaction rate increases, but the selectivity would decrease.^21^ As previously mentioned, during CO_2_ electrolysis for C_2_H_4_ production, the generation of CO must occur first. Thus, the use of His-loaded Cu_2_O electrocatalysts can be anticipated to improve CO generation by offering a larger surface due to the spherical shape of the particles and reduced particle size. Furthermore, loading His into Cu_2_O particles contributed to stabilizing CO on the electrode, facilitating the production of C_2_H_4_ instead of CO generation as the reaction progresses.

**Fig. 5:**
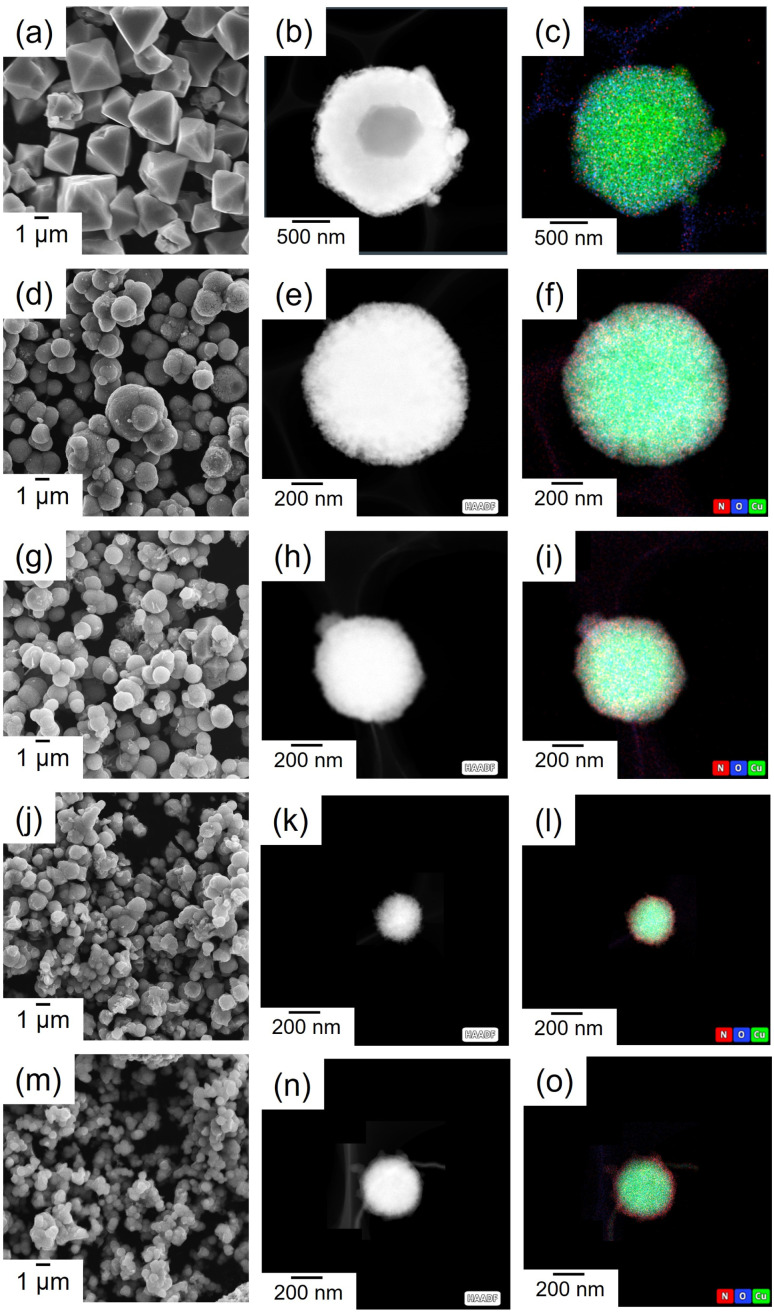
SEM images, HAADF-STEM images, and EDS mappings (Cu: green, O: blue, N: red) of the Cu_2_O particles synthesized with (a–c) 0, (d–f) 2.0, (g–i) 5.0, (j–l) 10.0, and (m–o) 20.0 mmol dm^−3^ His.

**Fig. 6:**
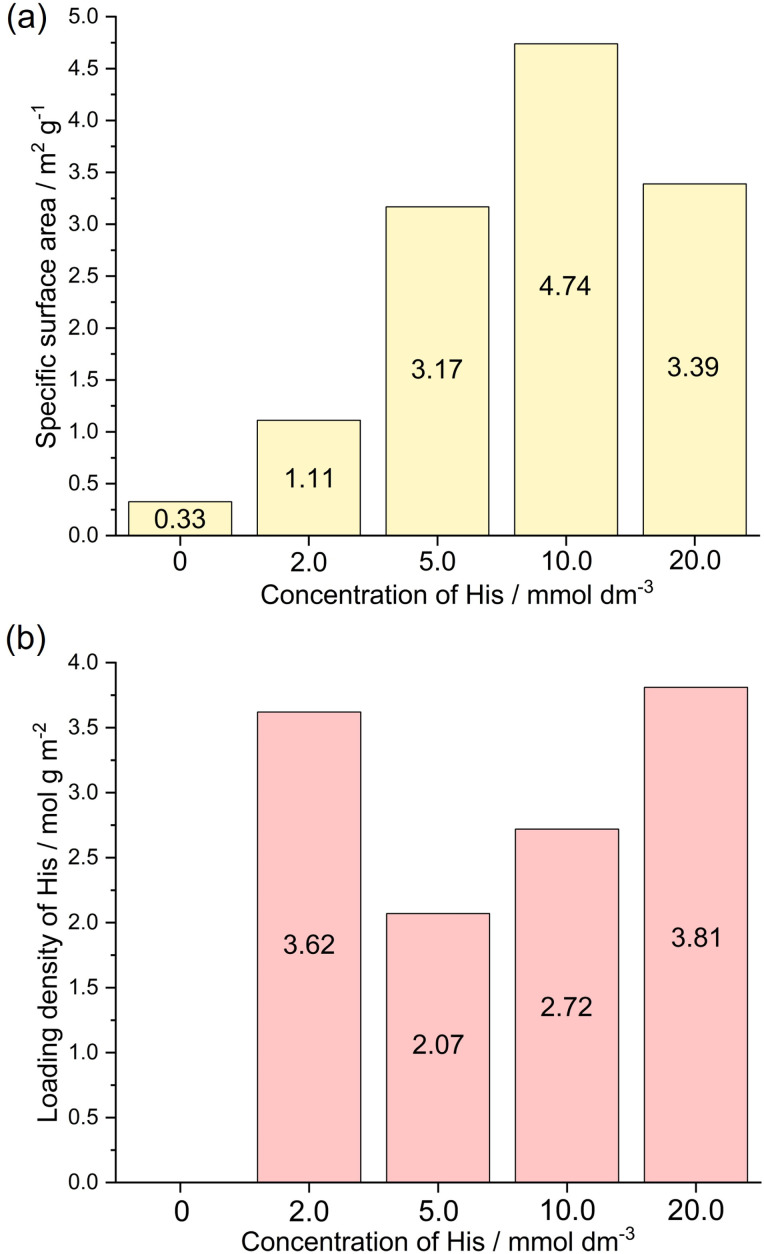
(a) Specific surface area and (b) calculated loading density of the Cu_2_O particles synthesized with 0, 2.0, 5.0, 10.0, and 20.0 mmol dm^−3^ His.

The synthesized His-loaded Cu_2_O hybrid particles, which exhibited the highest selectivity toward C_2_H_4_ production among the studied catalyst and a dramatically different morphology compared to that of None, were further examined. To investigate the effect of His concentration in the synthesizing precursor of Cu_2_O on the production and selectivity during CO_2_ electrolysis, the concentrations of His were varied from 0, 2.0, 5.0, 10.0, and 20.0 mmol dm^−3^. The concentration of 0 mmol dm^−3^ refers to the None. As the concentration of His increases, the intensity of the Cu_2_O-related XRD peak decreases, the peak becomes broader (Fig. S5a†), and the average crystallite size decreases (Fig. S5b†). The SEM images demonstrating surface morphologies, along with HAADF-STEM images and EDS mappings of Cu_2_O particles synthesized with different concentrations of His are illustrated in Fig. 5. The shape of the Cu_2_O particles clearly changes from octahedral (Fig. 5a) to spherical after adding 2.0 mmol dm^−3^ His (Fig. 5d). Furthermore, HAADF-STEM images demonstrate that the particle size decreases as the concentrations of His increases up to 10.0 mmol dm^−3^ (Fig. 5k) and remains the same or becomes slightly larger at 20.0 mmol dm^−3^ (Fig. 5n). The particle size reaches approximately 200 nm for His concentrations below 10.0 mmol dm^−3^. Certain amino acids function as capping agents in metal nanoparticle synthesis, enhancing dispersion and contributing to stabilization. Additionally, previous reports suggest that increasing the concentration of amino acids leads to smaller metal nanoparticle sizes.^17^ A similar phenomenon can be anticipated to occur during the synthesis of amino acid-loaded Cu_2_O particles in this study. The correlation between particle size and specific surface area was confirmed *via* BET analysis. The specific surface area values of His-loaded Cu_2_O particles synthesized with 0, 2.0, 5.0, 10.0, and 20.0 mmol dm^−3^ His show 0.33, 1.11, 3.17, 4.74, and 3.39 m^2^ g^−1^, respectively. These results demonstrated that a decrease in particle size leads to an increase in specific surface area (Fig. 6a). The presence of N atoms can be observed in the EDS maps obtained from the synthesized His-loaded Cu_2_O powders. Indeed, the presence of N atoms cannot be observed in the EDS maps obtained from 0 mmol dm^−3^ His. The presence or absence of N atoms can be clearly observed by comparing the EDS spectra of Cu_2_O particles synthesized with 0 mmol dm^−3^ and 10.0 mmol dm^−3^ His (Fig. S6†). In the EDS maps shown in Fig. 3f, i, l, and o, N atoms are primarily present on the surface of Cu_2_O particles, and the intensity of the N-related signal increases with the concentration of His. Thermogravimetric (TG) analysis demonstrated that the weight loss increased upon adding up to 10.0 mmol dm^−3^ of His and remains almost constant upon adding His at concentrations 10.0 and 20.0 mmol dm^−3^ (Fig. S7†). Based on the weight loss and specific surface area of each particle, His loading density of His-loaded Cu_2_O particles synthesized with 0, 2.0, 5.0, 10.0, and 20.0 mmol dm^−3^ were calculated as 0, 3.62, 2.07, 2.72, and 3.81 mol g m^−2^, respectively (Fig. 6b). The loading density of His did not exhibit any direct correlation with the concentration of His added to the precursor. The loading density of His decreases at 5.0 mmol dm^−3^ and increases thereafter. FT-IR spectra of the His-loaded Cu_2_O particles and a commercial His powder are shown in Fig. S8.† The incorporation of His to obtain the His-loaded Cu_2_O powders results in two new FT-IR peaks at approximately 1000 and 1500 cm^−1^ (indicated using yellow dashed circles in Fig. S8†), possibly originating from the loaded His. The FT-IR peak at approximately 600 cm^−1^ can be attributed to Cu_2_O (black dashed circle in Fig. S8†). For checking whether Cu_2_O particles can simply adsorb His on their surfaces, a soaking test was conducted by immersing None in a 10.0 mmol dm^−3^ aqueous solution of His for 60 min. Fig. S9† illustrates the FT-IR spectra recorded on the Cu_2_O particles before and after the soaking His aqueous solutions. In Fig. S9,† peaks at approximately 1000 and 1500 cm^−1^ are absent, confirming that the observed new peaks in the FT-IR spectrum of His-loaded Cu_2_O hybrid particles originate from His loading (Fig. S8†). In conclusion, His does not simply adsorb onto the surfaces of Cu_2_O particles by just soaking His solutions. The amino acid loaded-Cu_2_O hybrid particles can only be obtained by adding amino acids to the precursor solution used for Cu_2_O synthesis.

**Fig. 7:**
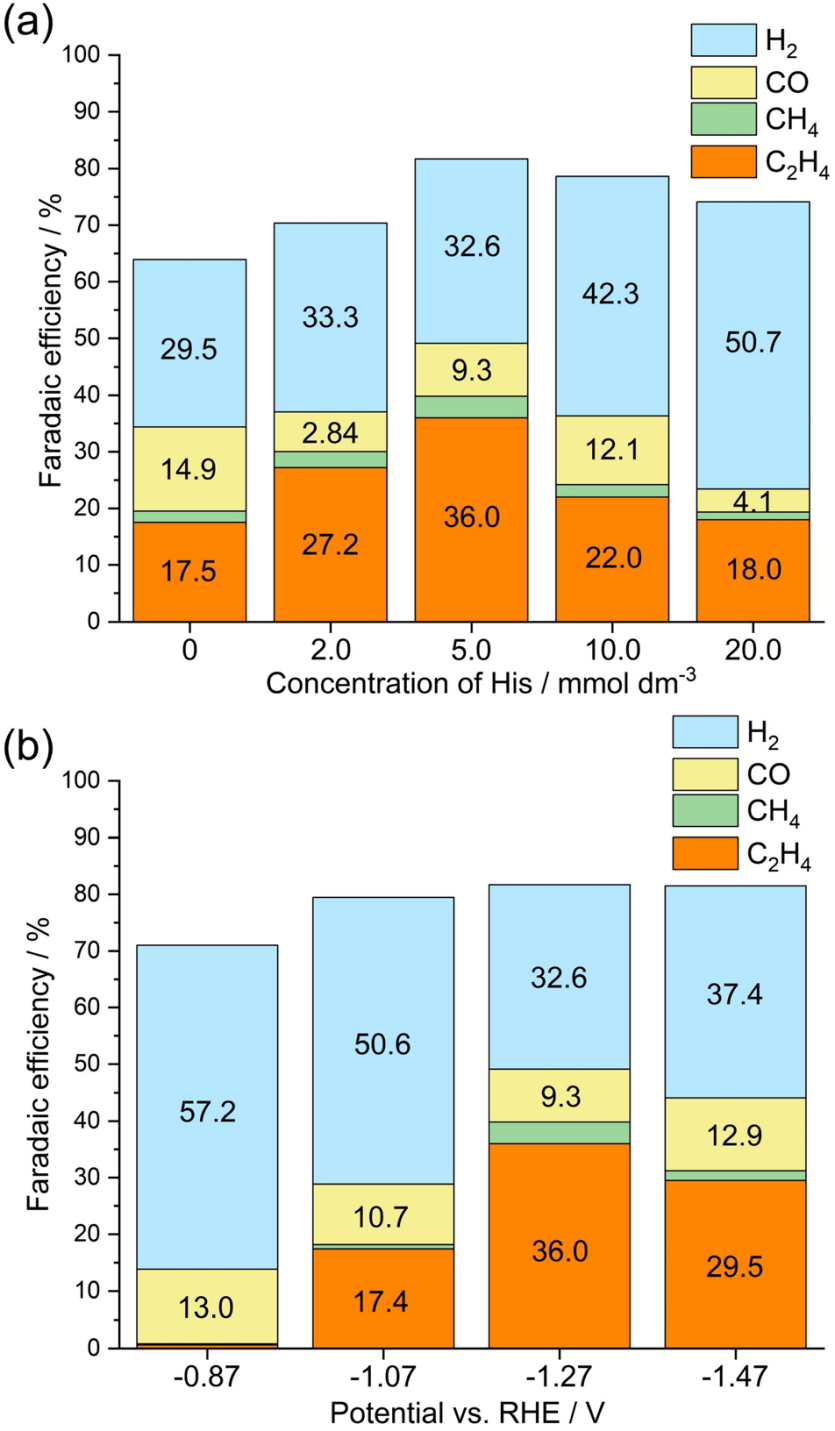
(a) FE as a function of His concentration exhibited by the His-loaded Cu_2_O electrocatalysis toward gaseous products during CO_2_ electrolysis under an applied potential of −1.27 V *vs.* RHE. (b) FE as a function of electrode potential exhibited by the His-loaded Cu_2_O electrocatalysis synthesized with 5.0 mmol dm^−3^ His toward gaseous products during CO_2_ electrolysis at 3.0 C in a CO_2_-purged 0.5 mol dm^−3^ aqueous KHCO_3_ solution (pH ≈ 8.75).

Fig. 7a illustrates the calculated FE values toward the formation of gaseous products during CO_2_ electrolysis in the presence of the His-loaded Cu_2_O electrocatalyst synthesized with 0, 2.0, 5.0, 10.0, and 20.0 mmol dm^−3^ of His at −1.27 V *vs.* RHE in a CO_2_-purged 0.5 mol dm^−3^ aqueous KHCO_3_ solution (pH ≈ 8.75). Fig. 7a shows that the FE value toward C_2_H_4_ production increases from 17.5% (for unloaded Cu_2_O) to 27.2% (for His loaded-Cu_2_O), even for the electrode based on Cu_2_O synthesized with 2.0 mmol dm^−3^ His. The FE value toward C_2_H_4_ production further increases to 36.0% for the synthesized with 5.0 mmol dm^−3^ His. However, for synthesized with 10.0 mmol dm^−3^ His, H_2_ production becomes dominant, decreasing the FE value toward C_2_H_4_ production to 22.0%, which further decreases to 18.0% for the synthesized with 20.0 mmol dm^−3^ His. Based on Fig. 6b, the amount of His loading onto Cu_2_O particles can be considered to have a stronger effect on C_2_H_4_ selectivity than the loading density of His. Excessive loading organics to electrode can be anticipated to completely cover the particle surface, inhibiting CO_2_ electrolysis. Fig. 7b illustrates the dependence of the FE values toward various products on the potential of the His-loaded Cu_2_O electrocatalyst synthesized with 5.0 mmol dm^−3^ His during CO_2_ electrolysis. At a potential of −0.87 V *vs.* RHE, C_2_H_4_ is not produced, and H_2_ is predominantly generated with an FE of 57.2%. As the potential changes to −1.07 V *vs.* RHE, C_2_H_4_ and H_2_ are produced with FE values of 17.4 and 50.6%, respectively. At a more negative potential of −1.27 V *vs.* RHE, C_2_H_4_ is produced with an increased FE of 36.0%, and H_2_ is produced with a reduced FE of 32.6%. However, at a more negative potential of −1.47 V *vs.* RHE, C_2_H_4_ is produced with a decreased FE of 29.5%, and H_2_ is produced with an increased FE of 37.4%, indicating that at an excessively negative potential, the competing HER dominates over the CO_2_RR. For simply checking the stability of the His-loaded Cu_2_O electrode synthesized with 5.0 mmol dm^−3^ His, CO_2_ electrolysis was conducted for 1800 s. In Fig. S11,† the chronoamperogram obtained during electrolysis demonstrates no sudden fluctuations in current and no catalytic detachment during or after electrolysis. We are currently undergoing not only an appropriate durability test by gas diffusion electrolysis with an online gas chromatogram but also computational science for understanding catalytic mechanisms on His-loaded Cu_2_O electrocatalysis during CO_2_ electrolysis.

## 4 Conclusions

In summary, we synthesized different amino acid-loaded Cu_2_O hybrid particles *via* microwave-assisted hydrothermal synthesis and evaluated their selectivities toward the formation of gaseous products during CO_2_ electrolysis. The amino acids were loaded into the Cu_2_O particles during synthesis. Notably, the His-loaded Cu_2_O hybrid particles exhibited a unique behaviour in which the particle shape changed drastically, with the particles exhibiting a significant reduction in their size. Furthermore, the His-loaded Cu_2_O based electrocatalysts synthesized with 5.0 mmol dm^−3^ His demonstrated an 18.5% increment compared to His-unloaded Cu_2_O toward C_2_H_4_ production during CO_2_ electrolysis. The loading of amino acids to Cu_2_O particles improved product selectivity, confirming that the proposed synthetic system is an effective catalyst for performing CO_2_ electrolysis.

## Data availability

The data supporting this article have been included as part of the ESI.†

## Author contributions

Y. Tsuda: conceptualization, data curation, funding acquisition, methodology, resources, supervision, writing − original draft, writing − review & editing; M. Irizawa: investigation, methodology; S. Fukuma: investigation, writing − review & editing; M. Kato: investigation, writing − review & editing; T. Gunji: investigation, writing − review & editing; K. Yoshii: investigation, writing−review & editing; N. Takeichi: funding acquisition, supervision, resources.

## Conflicts of interest

There are no conflicts to declare.

## Supplementary Material

RA-015-D5RA02252E-s001
