# Supplementary material for: Microwave-assisted hydrothermal synthesis of amino acid-loaded Cu2O hybrid particles for CO2 reduction electrocatalysis
Source: RSC Adv. 2025 May 15;15(20):16211–8. doi: 10.1039/d5ra02252e (PMC12079362; doi:10.1039/d5ra02252e)
Supplement: RA-015-D5RA02252E-s001 [file RA-015-D5RA02252E-s001.pdf]

Supplementary Information (SI) for RSC Advances.
This journal is © The Royal Society of Chemistry 2025

Supplementary information

Microwave-Assisted Hydrothermal Synthesis of Amino Acid-Loaded Cu_2O Hybrid Particles for CO_2 Reduction Electrocatalysis

Yuki Tsuda,[a,b*] Mizuki Irizawa,[c] Saki Fukuma,[a] Minami Kato,[a] Takao Gunji,[c] Kazuki Yoshii,[a] and Nobuhiko Takeichi[a]

[a] Research Institute of Electrochemical Energy, Department of Energy and Environment, National Institute of Advanced Industrial Science and Technology (AIST), Ikeda, Osaka 563-8577, Japan
[b] Renewable Energy Research Center, Department of Energy and Environment, National Institute of Advanced Industrial Science and Technology (AIST), 2-2-9 Machiikedai, Koriyama, Fukushima 963-0298, Japan
[c] Department of Chemical and Environmental Engineering, The University of Kitakyushu, Kitakyushu, Fukuoka 808-0135, Japan

*Corresponding author: y-tsuda@aist.go.jp (Y. T.)

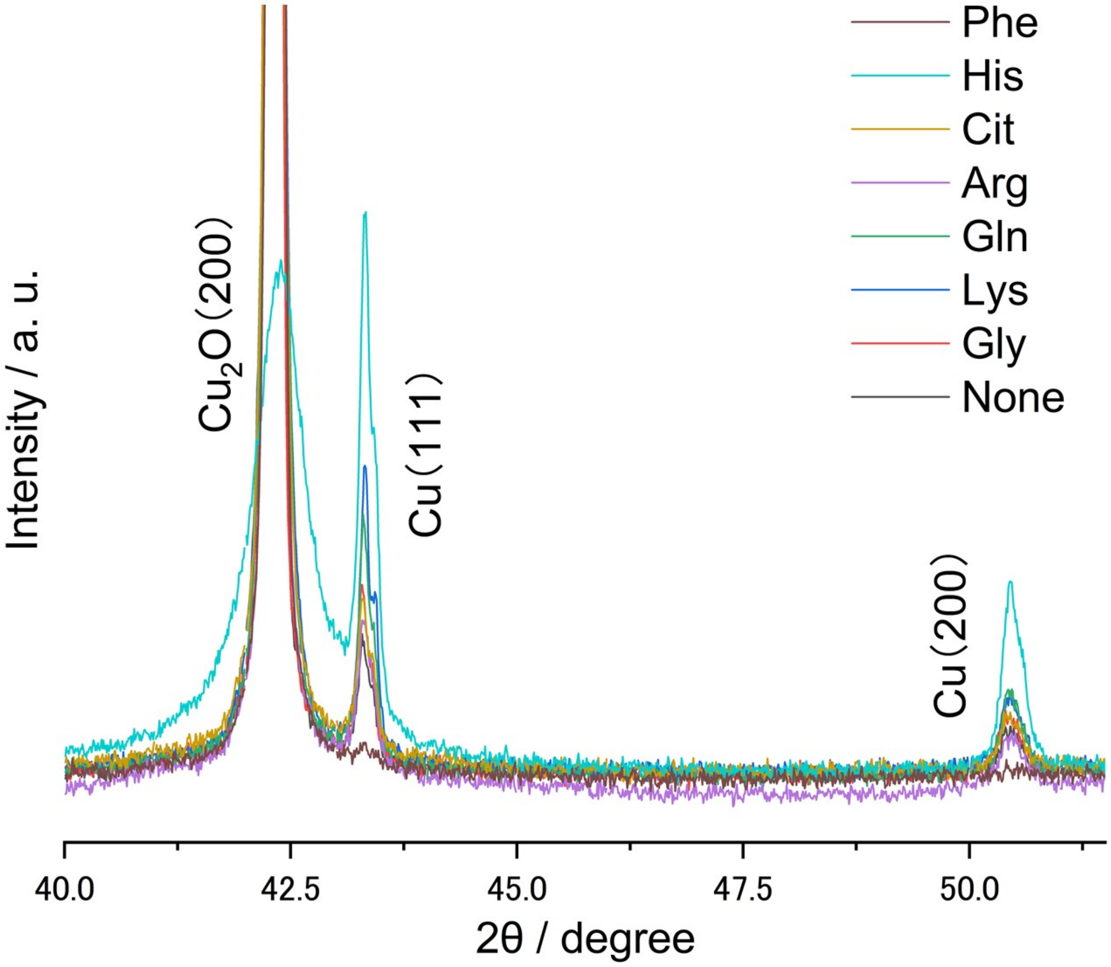

Fig. S1 Zoom-in XRD patterns in a 2θ range of 40.0–51.5°.

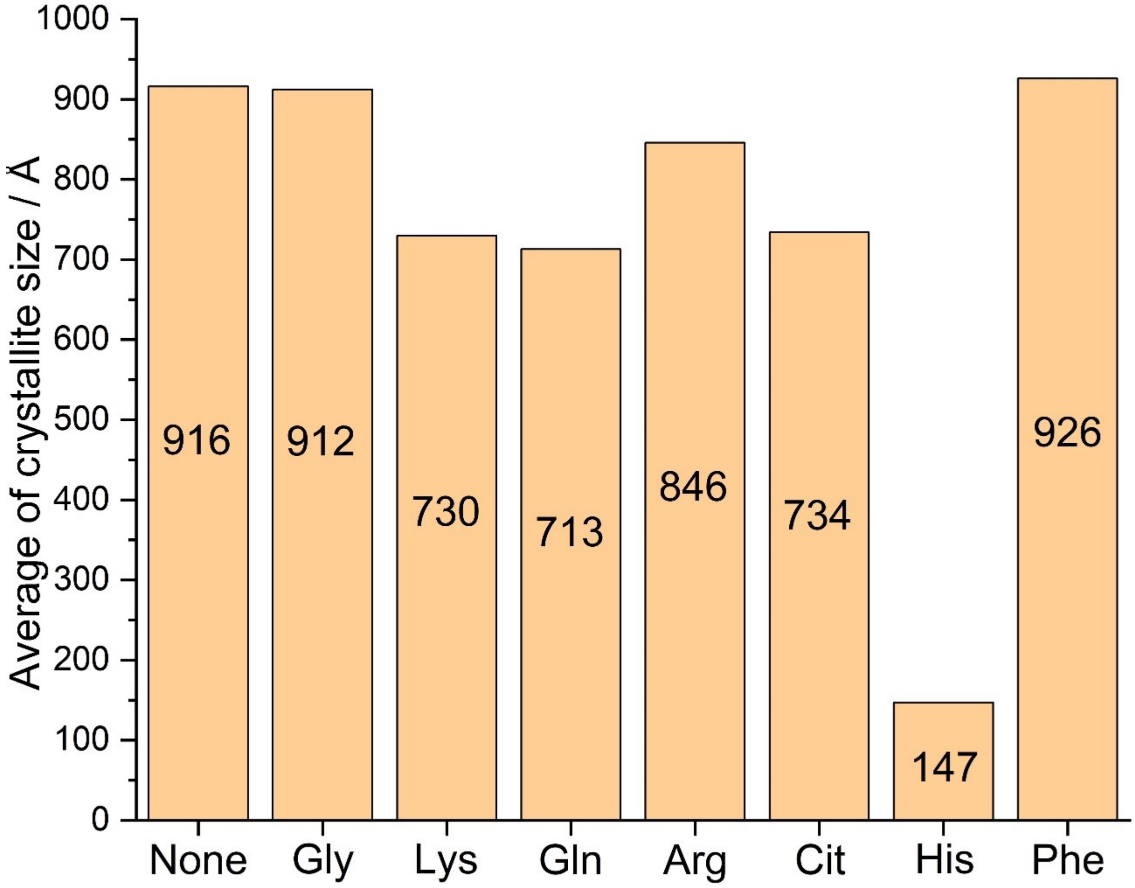

Fig. S2 Average crystallite size calculated by applying Scherrer equation for Cu_2O (111) diffraction peak of the synthesized Cu_2O with and without 5.0 mmol dm^{-3} amino acids.

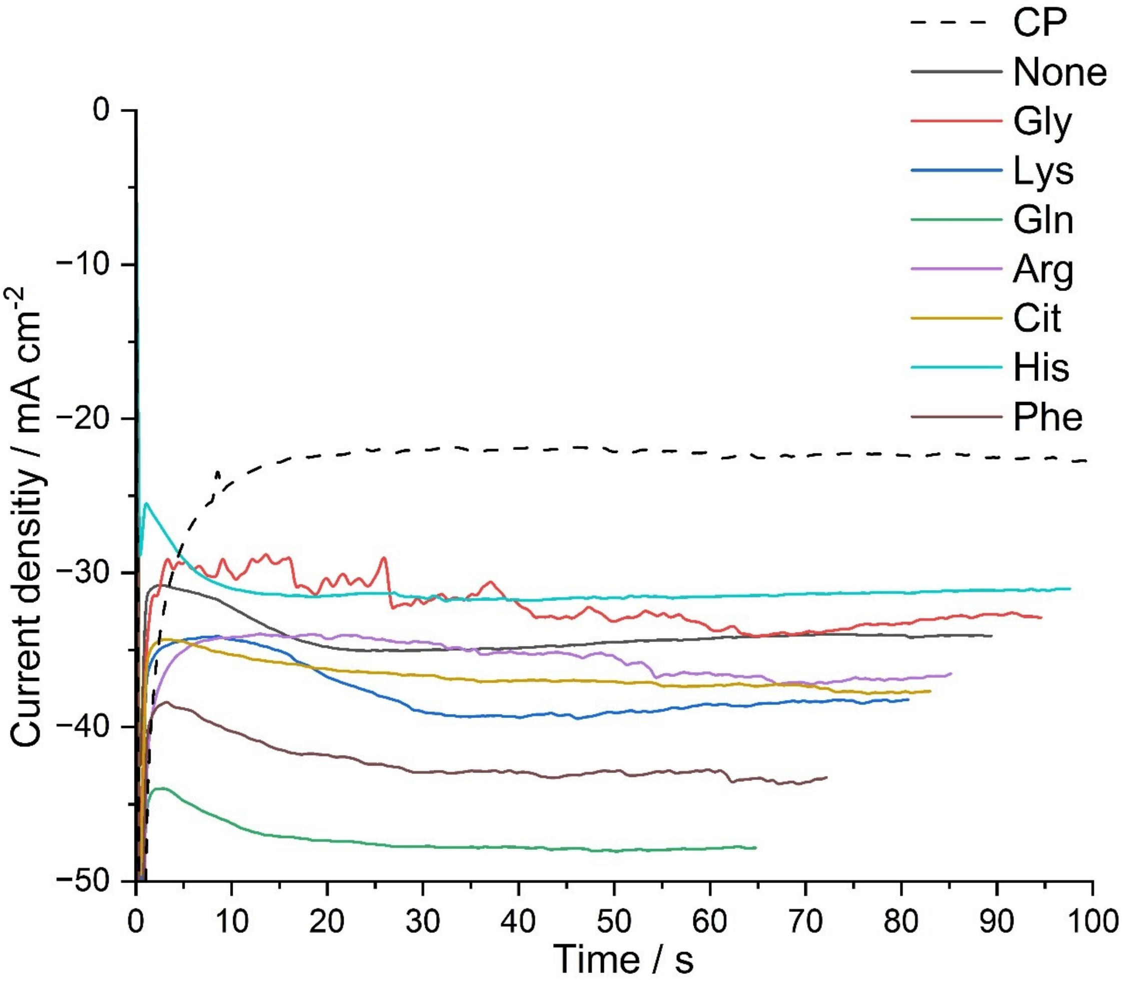

Fig. S3 Chronoamperogramas measured during CO_2 electrolysis under an applied potential of −1.27 V vs. RHE at 3.0 C in a CO_2-purged 0.5 mol dm^{-3} aqueous $KHCO_3$ solution (pH ≈ 8.75) using unloaded and amino acid-loaded Cu_2O electrodes synthesized with 5.0 mmol dm^{-3} amino acids and CP with Nafion ionomer.

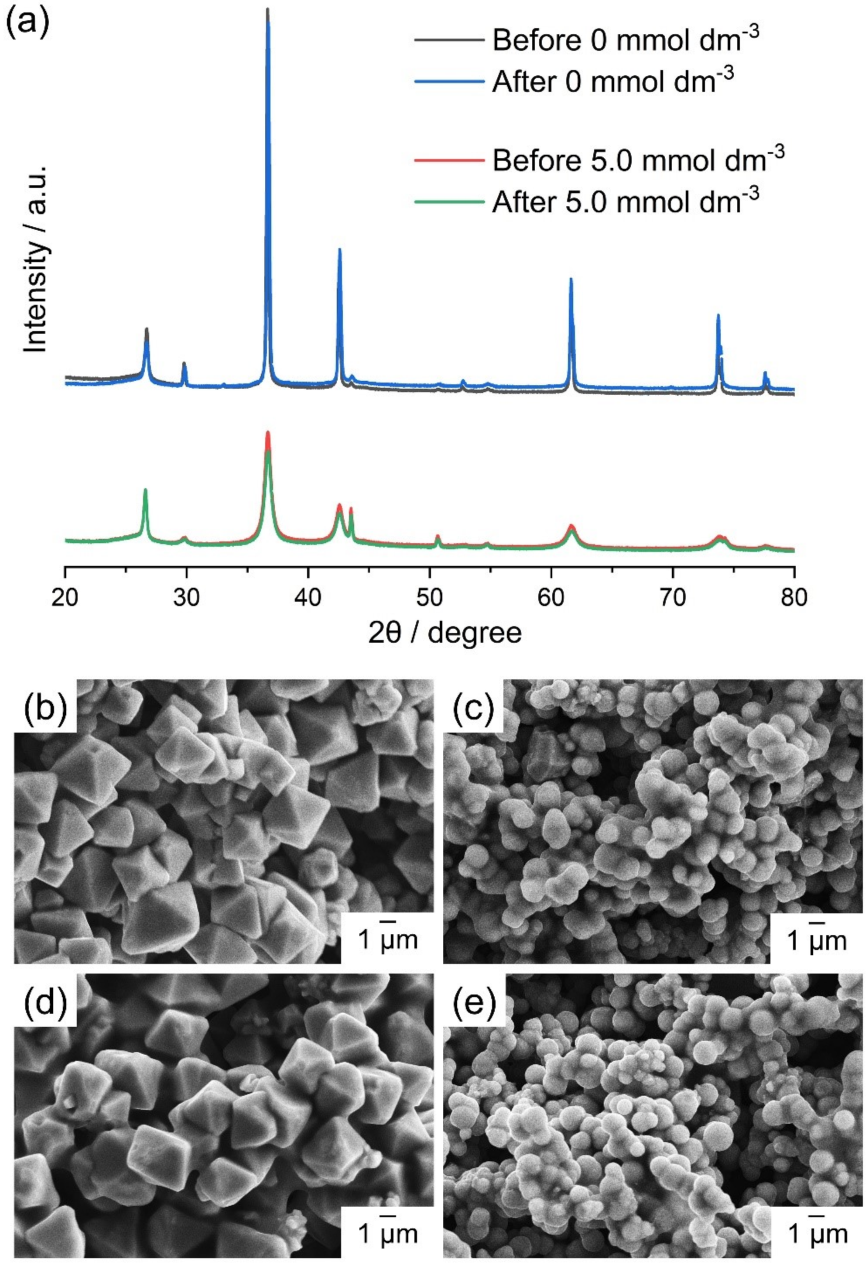

Fig. S4 XRD patterns (a) and SEM pictures of fabricated electrodes applied unloaded (b, d) and His-loaded Cu_2O particles synthesized 5.0 mmol dm^{-3} His (c, e) before (b, c) and after (d, e) CO_2 electrolysis.

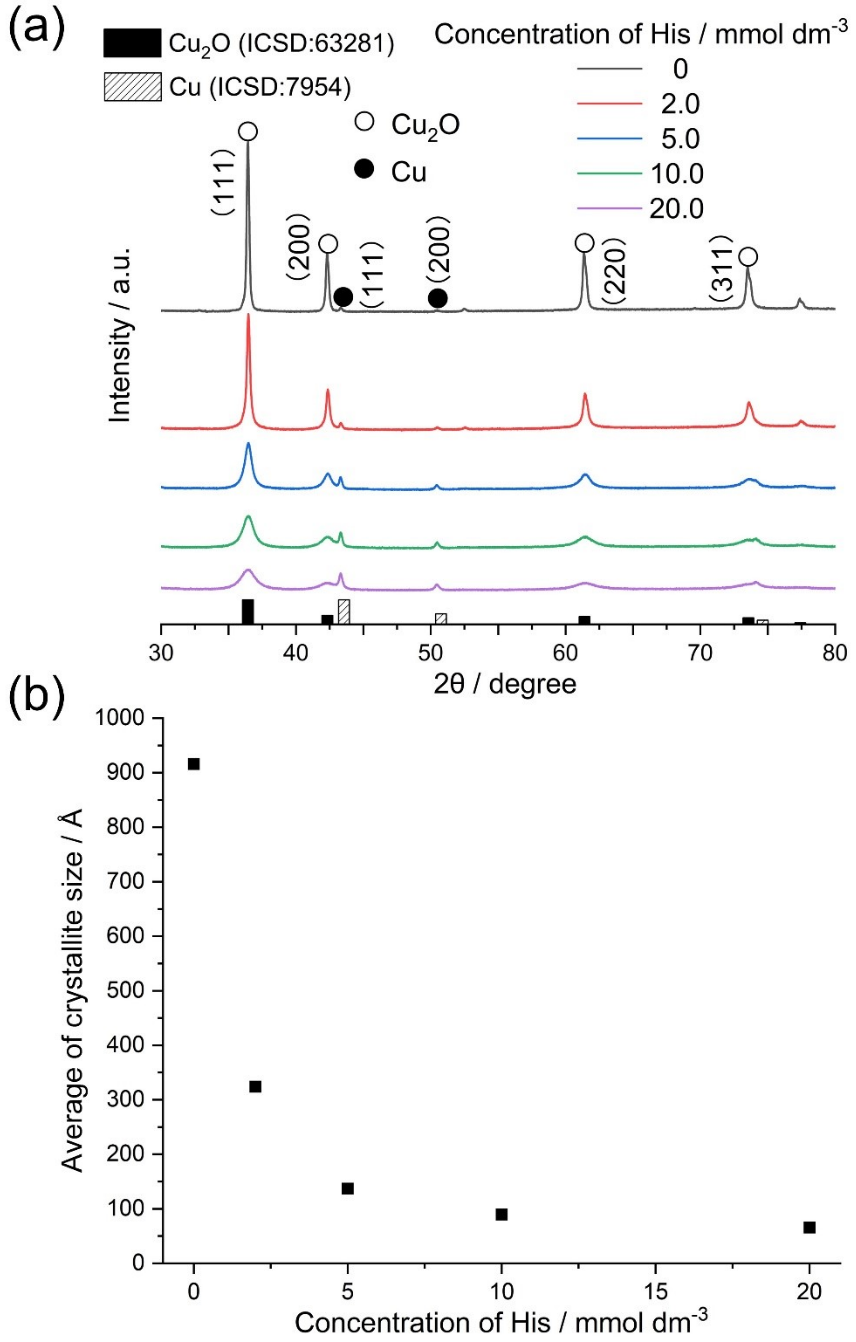

Fig. S5 XRD patterns and average of crystallite size of the His-loaded Cu_2O hybrid partiles synthesized with 0, 2.0, 5.0, 10.0 and 20.0 mmol dm^{-3} of His.

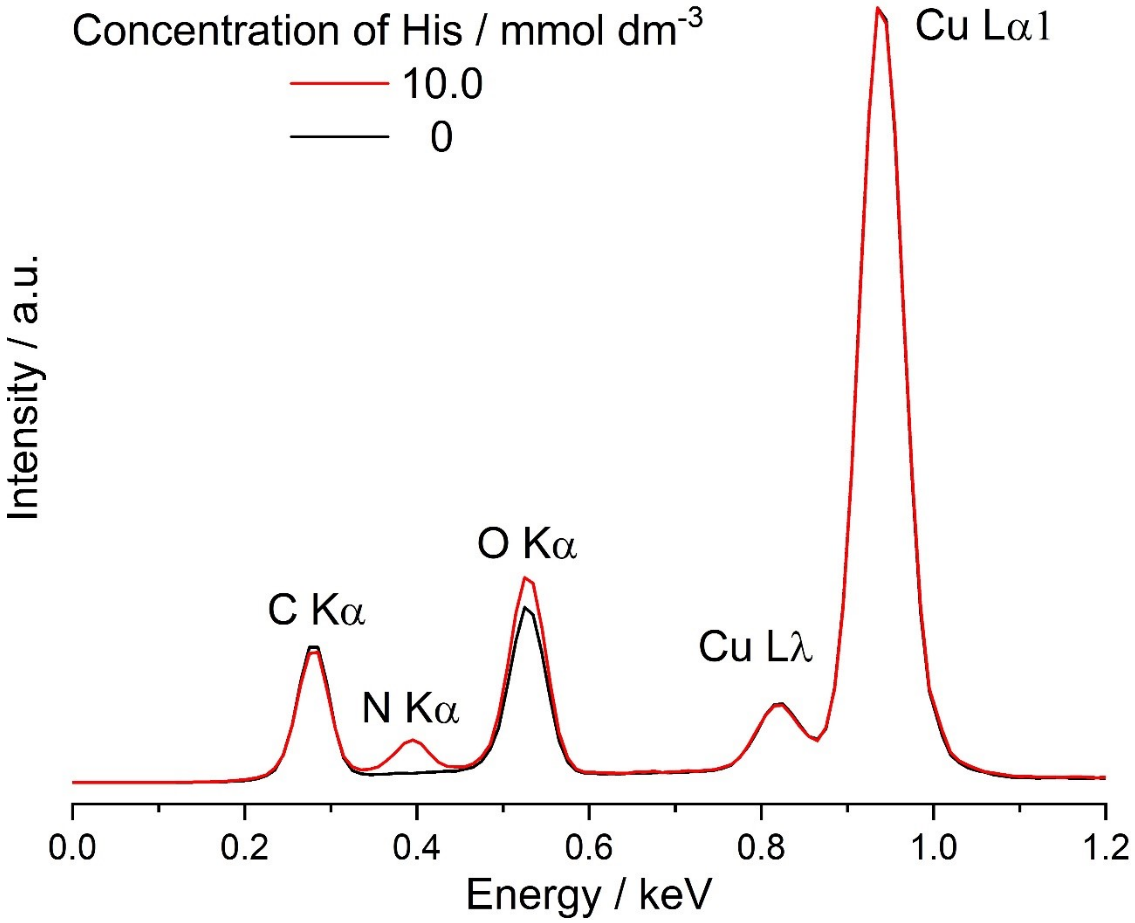

Fig. S6 EDS spectra of His-loaded Cu_2O hybrid particles synthesized with 0 and 10 mmol dm^{-3} L-His. Spectra were normalized to the intensity of the Cu Lα1 peak.

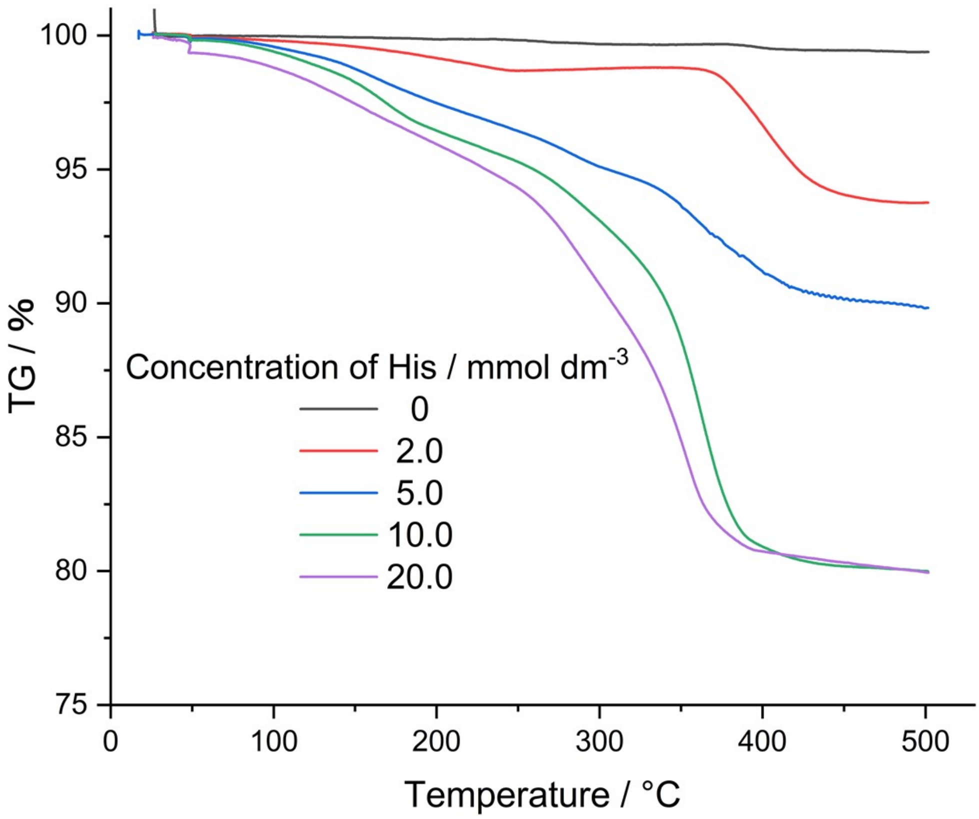

Fig. S7 TG curves of His-loaded Cu_2O hybrid particles synthesized with 0, 2.0, 5.0, 10.0 and 20.0 mmol dm^{-3}.

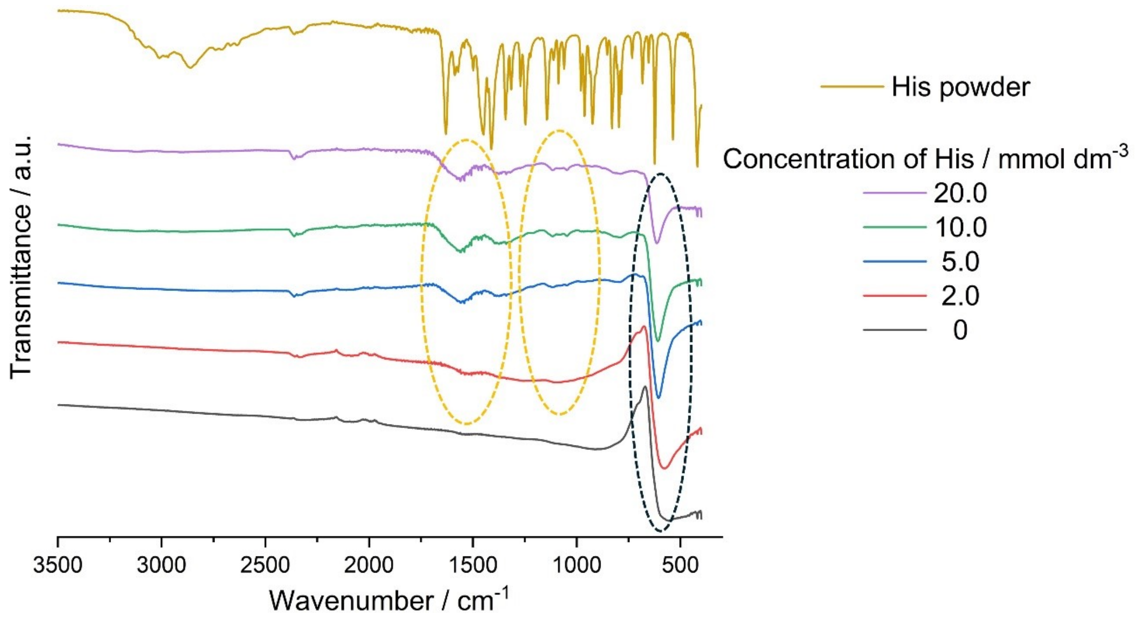

Fig. S8 IR spectra of His-loaded Cu_2O hybrid particles synthesized with 0, 2.0, 5.0, 10.0 and 20.0 mmol dm^{-3} of His and commercializing His powder. Black dashed circle means originated to Cu_2O peaks and yellow ones are peaks appeared by loading His.

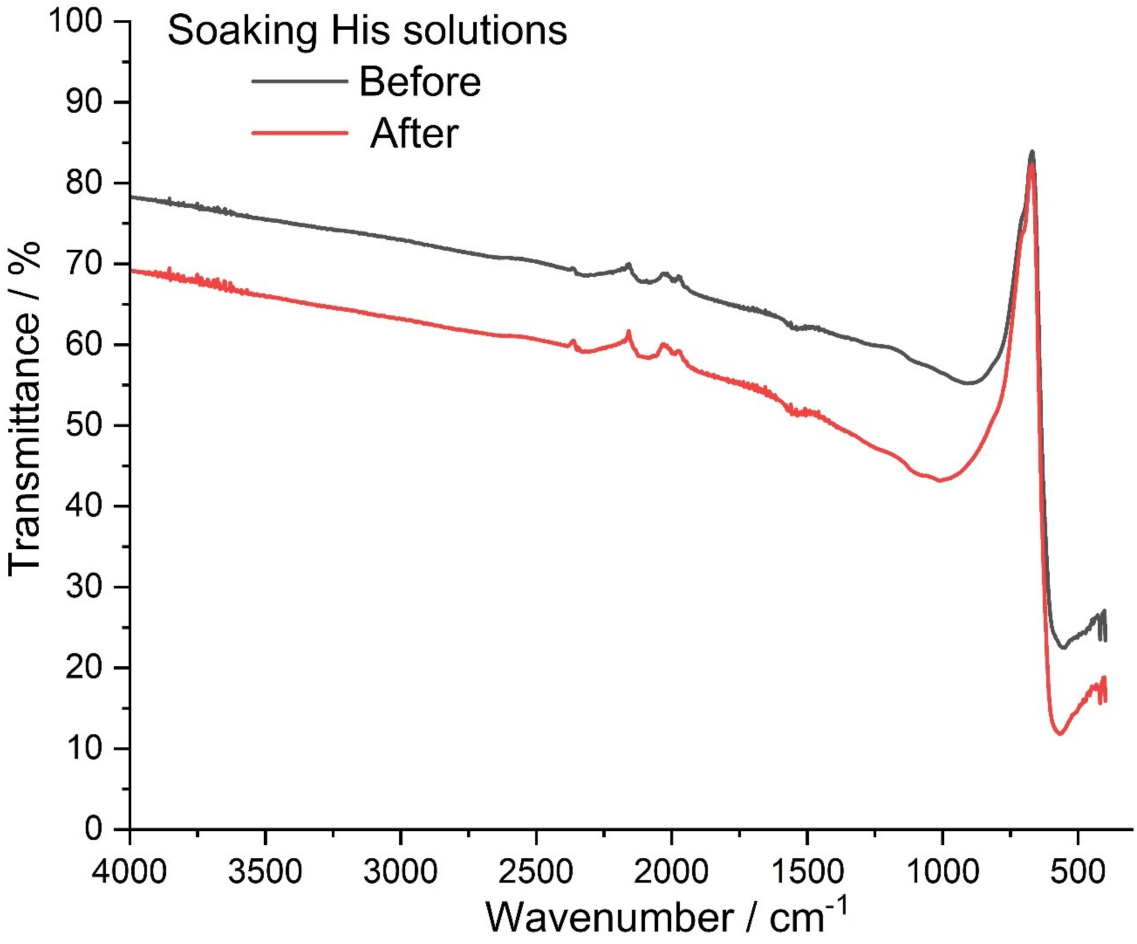

Fig. S9 FT-IR spectra of synthesized unloaded Cu_2O before and after soaking 60 min in the 10.0 mmol dm^{-3} His aqueous solutions.

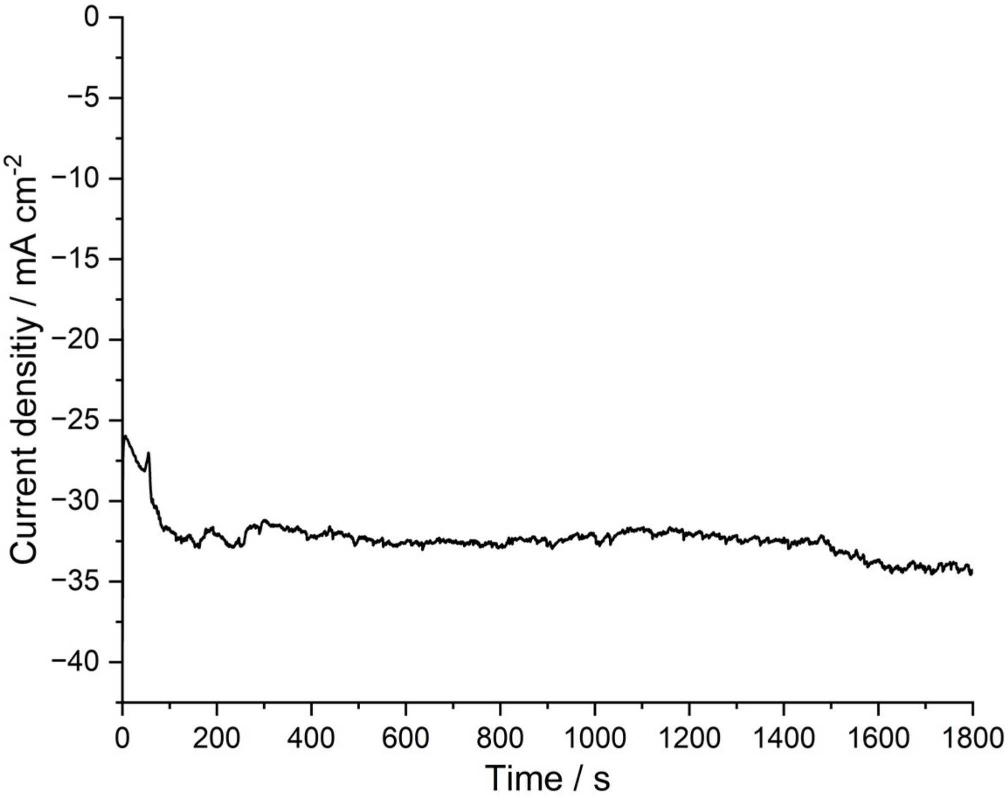

Fig. S10 Chronoamperogram during CO_2 electrolysis for 1800 seconds in a CO_2-purged 0.5 mol dm^{-3} aqueous $KHCO_3$ solution (pH ≈ 8.75) using His-loaded Cu_2Oelectrocatalysis synthesized with 5.0 mmol dm^{-3} His.
